# Pastoralists’ utilization and preferences for stakeholders and methods in livestock disease reporting and response in Northern Kenya: a participatory study

**DOI:** 10.1186/s12917-026-05286-1

**Published:** 2026-02-17

**Authors:** Derrick Noah Sentamu, Raphael Lotira Arasio, Dennis N. Makau, Joshua Orungo Onono

**Affiliations:** 1 Department of Public Health, Pharmacology and Toxicology, Faculty of Veterinary Sciences, University of Nairobi, P. O. Box 29053, Kangemi, 00625 Kenya; 2 Department of Land Resource Management and Agricultural Technology, Faculty of Agriculture, University of Nairobi, P. O. Box 29053, Kangemi, 00625 Kenya; 3 German Institute for Tropical and Subtropical Agriculture (DITSL), Steinstrasse 19, Witzenhausen, 37213 Germany; 4 Department of Biomedical and Diagnostic Sciences, University of Tennessee, Knoxville, TN 37996 USA

**Keywords:** Pastoral systems, Livestock, Participatory epidemiology, Disease surveillance

## Abstract

**Background:**

Livestock disease surveillance is important in early detection and control of diseases. In resource constrained settings, passive surveillance is predominately used, a system that relies heavily on the community to share information on livestock disease events for eventual response by relevant stakeholders. This study aimed to understand pastoralists’ utilization and reasons considered when choosing methods or stakeholders for reporting or responding to livestock disease occurrences in herds.

**Methods:**

The study was conducted in Marsabit county in Northern Kenya with pastoralists. Between August 2023 to August 2024, data was collected using participatory epidemiology tools including pairwise ranking and matrix scoring to profile the stakeholders and utilization of different methods with the respective reasons considered when choosing these for livestock disease reporting or response. Summary statistics from the output were presented in tables and graphs while the Kendall’s coefficient of concordance was used to show the level of agreement among various pastoralist groups/raters.

**Results:**

The disease reporting systems in Marsabit were most influenced by livestock owners (median rank = 9), friends and traditional healers (median rank = 7) and Elders’ council and Private AHWs (median rank = 6). Disease reporting was primarily through mobile phones (median rank = 4) with their usage increasing markedly between 2001 and 2024. Livestock disease response was most frequently offered by livestock owners, friends, and agrovets/private practitioners, with median ranks of 9, 7 and 6, respectively. The response to disease events in herds was mostly by pastoralists themselves, using synthetic drugs (median = 8) and this practice had increased overtime from before the 1980s to 2024. The pastoralists’ decisions to report a disease event were largely influenced by accessibility of the stakeholder or method of reporting, perceived technical knowledge of recipient, cost friendliness and affordability of the services, while ability to provide quick response, credit facilities for services, having technical knowledge and affordability were important reasons for their choice of the process of disease response.

**Conclusions:**

This study highlights the central role livestock owners’ play in disease reporting and response in underserved pastoralist areas of northern Kenya, with a limited role played by government animal health services providers. The results suggest that veterinary services delivery systems should be reviewed with input from community stakeholders to improve surveillance and disease reporting. This integration would enhance livestock disease surveillance and protect pastoralists’ livelihoods.

**Supplementary Information:**

The online version contains supplementary material available at 10.1186/s12917-026-05286-1.

## Introduction

The World Organization for Animal Health [1] defines a livestock disease surveillance system as the use of one or more surveillance components to generate information on the health status of animal populations. Passive surveillance, defined as the continuous collection and reporting of animal health information to authorities, is a widely utilized livestock disease surveillance component in resource constrained settings [2, 3]. Being a continuous and non-costly process, community level stakeholders like livestock owners, field Private Animal Health Workers (AHWs), Community Disease Reporters (CDRs), slaughterhouse operators, livestock traders and other stakeholders often share or report livestock disease information to authorities in expectation of response interventions. This is the case in the Arid and Semi-Arid Lands (ASALs) of Kenya [4, 5].

The ASALs of Kenya are estimated to cover 80% of the dry land mass, support 26–30% of the national population and 50–70% of the national livestock herd [6]. Pastoralism is the dominant economic activity in the ASALs with close to 9 million pastoralists managing over 70% of the country’s cattle, sheep, goats, donkeys and camels populations [7, 8]. Animal diseases are a major limitation to pastoral livestock production [9–11]. A high number of livestock are raised over extensive swathes of land, combined with their regular mobility creates profound challenges to an under resourced, national government reliant animal health service delivery system where the few AHWs in these regions are not readily facilitated for consistent active disease surveillance, let alone having ready access to state-of-the-art laboratory diagnostic services and infrastructure as would be desired [12–14]. Consequently, disease surveillance is largely passive in nature, relying heavily on pastoralists and other community members who are closer to animals to make clinical observations which they would report to authorities/technicians for eventual response [15]. However, there is still a gap in knowledge on how they make the decision on which stakeholder would be used for disease reporting and response and what reasons guide this decision-making process.

Despite the constraints for active disease surveillance systems in ASAL areas, limited government interventions have been made to enhance passive disease surveillance in those regions in Kenya. Notably, these have included incorporation of Community Based Animal Health Workers (CBAHWs), individuals who were trained by competent NGO and government veterinarians to conduct basic diagnoses, treatments and inform authorities on disease events [16–19]. CBAHWs have since been outlawed by the Kenya Veterinary Board and their important role of sharing disease events to government veterinarians digitized and outsourced to CDRs and Private Animal Health Workers [20–23]. However, there is a dearth in empirical evidence to support the efficient designing/development and mainstreaming of these interventions. Furthermore, there’s a paucity of studies describing operation of passive surveillance system, the stakeholders involved, flow of information and connectedness in disease reporting and response in pastoral areas [24, 25].

Participatory Epidemiology (PE) is a branch of pidemiology that employs the systematic use of participatory approaches and methods to improve the understanding of diseases and options for animal disease control [15]. PE is of particular importance in resource limited settings as it efficiently harnesses local stakeholders’ knowledge, lived experiences and motivations to adequately understand an existing situation and further gives them a greater role in designing appropriate interventions [26].

A diverse range of interviewing, scoring, ranking and visualization methods are used during the discourse with the community to produce both qualitative and quantitative output [27–29]. Because of their adaptability and flexibility, participatory methods have globally and widely gained traction for use in understanding various paradigms of animal health and services through the lens of local stakeholders [30]. Most importantly for this study, the use of PE provided the latitude to navigate the motivations, social and mental constructs around which pastoralists make decisions on livestock disease reporting and response through investigation of factors they considered in this process. For example, studies in Bolivia and Netherlands identified previous experience with a stakeholder, fear of negative consequences, guilt, shame and prejudice, conflict of interest with a stakeholder, procedural injustice after reporting, concern for ethical practice, trustworthiness and accessibility of the stakeholder as important factors for community members when making decisions on livestock disease reporting [31, 32]. An understanding of these, would provide some rigor, legitimacy and sensitivity to local perspectives and context in efforts of improvement of a surveillance system.

The aim of this study was to understand how pastoralists utilize different stakeholders and methods for livestock disease reporting and response and reasons they often considered when they make these choices. These preferences will be empirical reference to guide practitioners and decision makers in designing or improvement of locally relevant surveillance systems for pastoral settings.

## Methods

### Study area and sampling strategy

The study was conducted in Marsabit County, in Northern Kenya, purposively selected because all indigenous tribes in this county practice pastoralism as their way of life, providing the right sample pool to meet the objectives of this study. Most parts of the county are arid and most of the land is communally owned. Livestock are the major economic enterprise with the dominant production system being pastoralism. The main livestock products are milk, beef, chevon, mutton and camel meat [33].

The study area was Laisamis subcounty; Korr/Ngurnit and Laisamis wards, selected through a multistage random sampling approach for the larger project “Increasing efficiency in rangeland-based livestock value chains through machine learning and digital technologies” (InfoRange). InfoRange is a transdisciplinary project with an aim of co – designing a digital platform for improved livestock disease reporting and response in pastoral Kenya.

Laisamis subcounty is predominately occupied by *Rendille* and *Ariaal* pastoralists. The *Ariaal* are a community that emerged from the interdependence and cooperation of the *Samburu* and *Rendille* in an era where they shared common enemies, with alliances presently maintained through intermarriage and intermigrations. They speak a Nilotic language that is a combination of *Kisamburu* and the Cushitic *Kirendille*. The Rendille on the other hand are Somaloid peoples believed to have originally lived but later migrated westward from southern Ethiopia and Somalia to settle around Lake Turkana. They speak *Kirendille*, a Cushitic language closely related to the Somali. Both groups are nomadic pastoralists [34, 35]. The final study sites were sublocations from the wards. These were determined using simple random probability sampling. All sublocations were written on sheets of paper which were folded, shuffled and randomly selected from, returning nine study sites including Merille, Tirgemo, Sakardalla, Lokshura, Lmotit, Illaut, Orotilkes and Korr sublocations, where data was collected through a cross-sectional study design.

Data were collected from pastoralists, and the discussion groups consisted of men since they bear a central role of keeping, managing and decision making on livestock keeping both at household and community level. Their ages ranged between 23 and 80 years. Other stakeholder categories invited to the discussion groups had known active roles within the livestock disease reporting and response ecosystem. Therefore, a single group was ultimately constituted by an Elder, a Community Disease Reporter (CDR), a traditional healer and other community members.

### Study design

Stakeholders and methods used in livestock disease reporting and response within these pastoralist communities have previously been published [24]. Briefly, the stakeholders included community elders’ council, pastoralists’ friends, chiefs, livestock owners, agrovet/private animal health workers, traditional healers, radio, community disease reporters (CDR), elected politician (ward level) and government AHW. Livestock owners owned the animals and were the final decision makers on treatment or movement patterns of their animals. The disease reporting methods included: Walking (all kind of movement by foot), Motor vehicles, Radio, Phone and Motorbikes. Finally, methods used to respond to disease occurrences in these communities included mass treatment with synthetic medicines, mass vaccination, local advice, technical advice, routine treatment by private AHWs, routine treatment by government AHWs, self-management with synthetic drugs, self-management with alternative veterinary practices and management by traditional healers.

For this study, the identified stakeholders and methods for disease reporting and response were subjected to participatory epidemiology techniques as described in the proceeding sections of the methods, with the aim of understanding how pastoralists utilize them and how decision making happens as choices are made. Ranking and scoring methods were deployed to realize this objective. This study adopted a cross – sectional study design, carried out at a point in time.

### Data collection

**Fig. 1:**
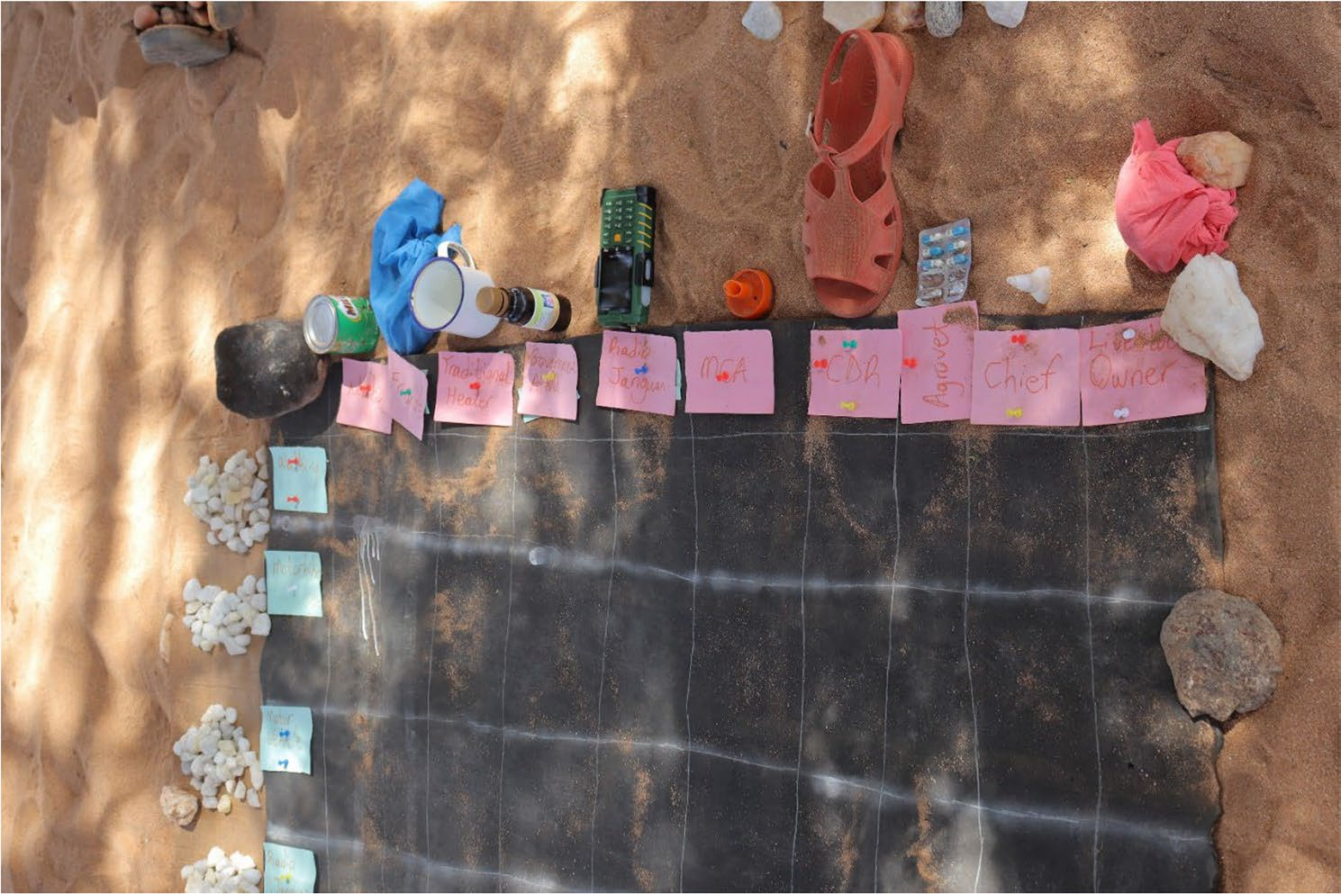
An image showing arrangement of the matrix and symbols during data collection

Through 27 Focus Group Discussions (FGDs), data were collected with the assistance of locally recruited Research Assistants who translated Kiswahili as spoken by the researcher to the local dialect of the *Rendille/Ariaal*. The researcher facilitated the discussions and was skilled in PE techniques. Participatory tools including pairwise ranking, matrix scoring and timelines with proportional piling were used. Each session began with an introduction of the items to be discussed and describing its respective participatory tool of utility, in a stepwise manner. Step 1: Participants were requested to select objects/symbols from the environment to associate with the items of discussion. The respondents understood and memorized the objects better than if plain English/*Rendille/Ariaal* word labels were used. For example, for stakeholders, they could choose a stone to represent a Traditional healer, other different symbols would be chosen to represent other stakeholders. For reporting methods, they could choose an old disposed of shoe to represent “Walking”, other appropriate objects would be chosen to represent all other reporting and response methods. Figure 1 below shows an example of how this was done. This simple exercise preceded each pairwise ranking, matrix scoring and timeline with proportional piling session. Step 2: The symbols would be respectively placed on the table axes and the participants re – acquainted with them to ensure they perfectly recalled what they represented before the questions were presented for discussion. Step 3: Due to weather constraints, tables were drawn on a black mat (instead of the ground).

The facilitator ensured that each of the participants had an opportunity to speak. Participants were given sufficient time to discuss and come to a consensus before they unanimously effected a given rank or score.

### Reasons considered when choosing a reporting or response method or stakeholder

Pairwise ranking activities were carried out where reasons why a stakeholder or method was chosen over the others were obtained. All stakeholders were placed in the same order on both x and y axes and each stakeholder compared against all others while asking why respondents would choose one over the other. This was done with the different groups of pastoralists in the different study sites until no newer reasons were mentioned (point of saturation). The same was done for the methods. All the reasons given are in Table 2. This activity was also important for determining which stakeholders/methods the pastoralists utilize most.

### Identification of memorable events for timelines with proportional piling activities

In the early stages of the work (June 2024), to identify memorable events in the lifetime of the pastoralists between 1980 and 2024, qualitative methods including narrative interviews and FGDs were used to engage the pastoralists. It was emphasized that these events should be known by the entire community. The most memorable event (mentioned/affirmed by many respondents as memorable) in a 10-year time period, was chosen to represent that time interval, as per the Gregorian calendar. This is important because our target population, the pastoralists, are often unable to pinpoint years but rather associate time periods with memorable events. These periods were important to facilitate usage of timelines with proportional piling technique. Table 1 shows the time periods and their representative and memorable events.

**Table 1:** A table showing time periods as related to memorable events in the *Ariaal* and *Rendille* communities

Time Period	Memorable Events	
	Ariaal	Rendille
1981–1990	*Aramia* (A period when violent Somali bandits rustled animals from Ariaal communities)	*Aramia* (A period when violent Somali bandits rustled animals from Rendille communities, they were also after elephant tusks and rhino horns)
1991–2000	*Loidikedike* (El Nino)	*El Nino* (El Nino)
2001–2010	*Serelpalwa* (The period that came immediately after El Nino associated with a place where communities were displaced to)	*Salmate lagube* (A period when a prominent grazing ground named “*Salmate”* was set on fire as a control measure against heavy infestation of ectoparasites and wild animals)
2011–2020	*Machiefi* (a period when local chiefs were killed along the Laisamis – Marsabit highway as they went to negotiate for peace with the Gabra tribe)	*Maanti Oor layigis* (A period when the Eastern and Western Rendille moieties/blocs disagreed concerning initiation ceremony arrangements, culminating into a prolonged local conflict)
2021 – date	*Taata* (Now)	*Tolla* (Now)

### Ranking of important species and its diseases, timelines with proportional piling and matrix scoring activities

A total of 27 focus group discussions averagely consisting of 7 participants, were held over a course of three days in each study site, each lasting approximately 3–4 hours. Further details on the participants can be found in Supplementary Material 2. A tool was developed to guide the discussions (Supplementary Material 1). It was pretested in Ngurnt sublocation with participants the study did not proceed with.

To determine the most important livestock species, participants began by listing the major livestock species in the region. Pairwise ranking was done to compare each species with all the others, a pair at a time. The most important species was the one that had the maximum number of counts from the pairwise ranking exercise. The participants were then requested to list the most important diseases affecting the most important species identified. Local terms describing the diseases by the pastoralists were cross validated with the locally available county veterinary handbooks used by the local AHWs [36, 37, 38] and other relevant literature [39, 40] that have the complementary English names of the terms. Further validation was done through follow up calls to the local AHWs. Each of the diseases was compared with all the others a pair at a time using pairwise ranking. Information of these diseases was important primarily for scenario/stratified analysis through matrix scoring. Here, the diseases were placed on the y – axis of the matrix while the stakeholders/methods on the x – axis, the participants would then be asked to use stones to relatively score which stakeholders/methods they utilize most for reporting/response of a given disease.

Timeline with proportional piling was carried out to determine the utilization of stakeholders and methods overtime, where events (time periods) as shown in Table 1 were placed on the x – axis and stakeholders/methods on the y – axis and scoring was done using 100 stones, reflecting the relative use of each stakeholder/method in each time period.

Matrix scoring was used to score reporting and response stakeholders/methods alongside the important factors considered as listed in Table 2.

**Table 2:** Reasons considered by pastoralists when choosing stakeholders or methods for disease reporting or response

Reasons influencing choice of stakeholders utilized in disease reporting
1. Sympathetic to one’s situation 2. Easily accessible because of proximity to community 3. Easily accessible on phone 4. More Knowledge and expertise in synthetic medicine and its utilization 5. Can spread information wide 6. Understands you when you explain to them 7. Can support with own resources to facilitate reporting e.g. giving you airtime for you to call
Reasons influencing choice of stakeholders utilized in disease response
1. Offers quicker response 2. With special indigenous knowledge (e.g. fracture management, assisted delivery) 3. Can consult others if they do not know the remedy 4. Can offer services on credit 5. Can support with own resources to facilitate response e.g. giving an animal to sell to buy drugs
Reasons influencing choice of methods utilized in reporting a disease
1. Affordability 2. Fast spread of information (it can spread the information faster) 3. Can be shared (with neighbors/friends when reporting) 4. Spreads information wide 5. Easily accessible/available 6. Method is within the livestock keeper’s control
Reasons influencing choice of methods utilized in responding to a disease
1. Affordability 2. The method covers many herds (benefits many livestock keepers) 3. Provides quicker response 4. Based on indigenous knowledge (local remedies) 5. Needs the knowledge of a technician

### Data management and analysis

**Fig. 2:**
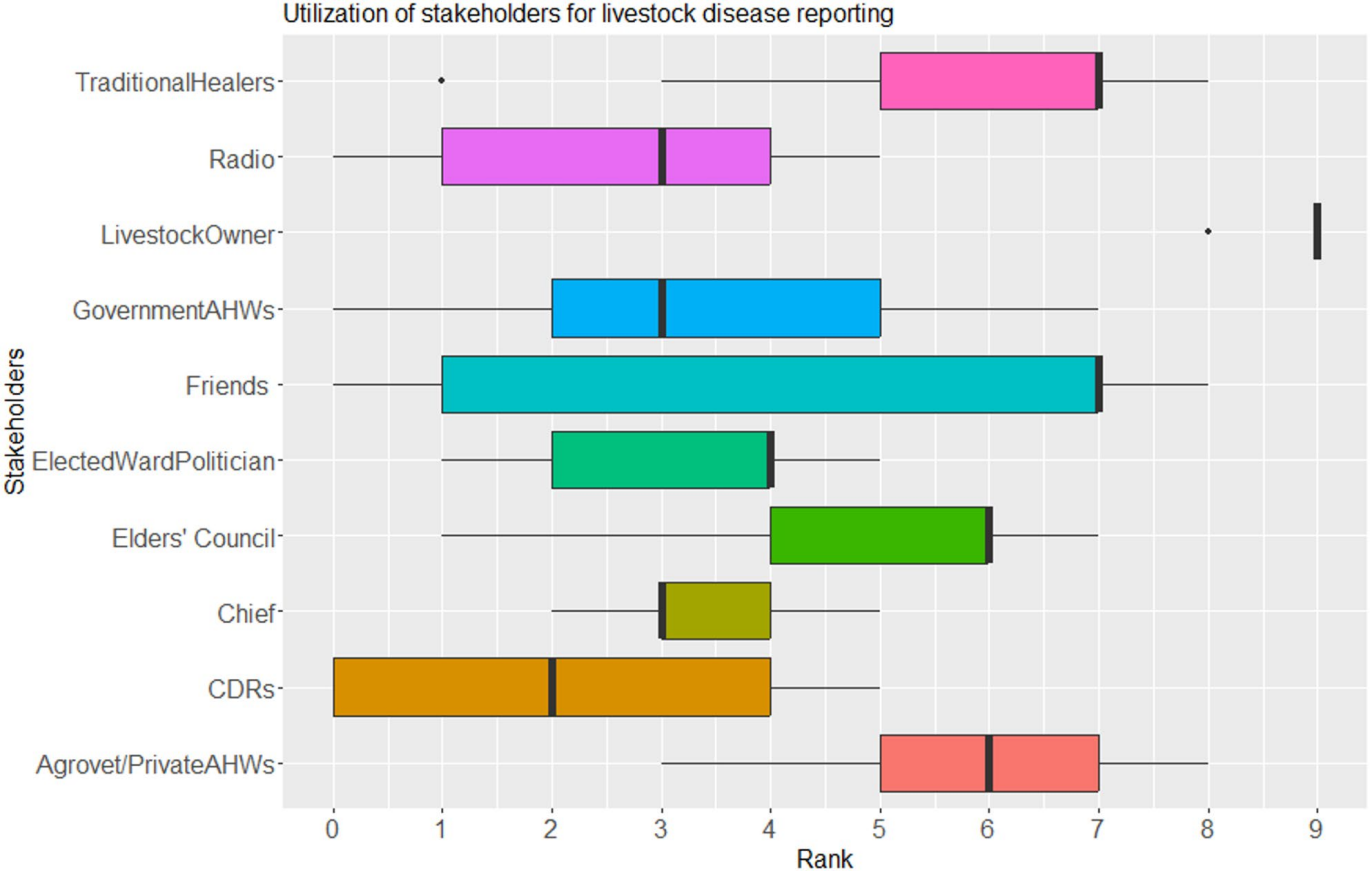
Rankings for stakeholders as utilized for livestock disease reporting in Laisamis Sub-county, Kenya

Comprehensive notes were taken during the sessions. The data were entered in Microsoft Excel software and appropriately cleaned. Microsoft Excel and R statistical software [41] were used for data analysis. For pairwise ranking, the number of times a given item was selected over the others was counted and recorded as the ranking of that item. Descriptive statistics including the median and graphical displays were used to represent this data. In Figs. 2, 5 and 7, the black lines in the box and whisker plots represent the medians. For timelines with proportional piling, the median scores were obtained and used to plot graphs showing the trends. For matrix scoring, a summarized matrix that captured scores from all groups were drawn. Inferential analysis for matrix scoring was done using the Kendall’s coefficient of concordance (W); a non – parametric test that measures association between sets of ranks and a measure of inter-rater reliability (in the context of this study, the agreement between different groups), (W), that was between 0 and 1, with a corresponding p –value [42]. All matrices are guided and interpreted as follows: no * *p* value > 0.05, *p value < 0.05, **p value < 0.0005, W < / = 0.3 (Weak), W > 0.3 < 0.5 (Moderate), W > 0.5 (Strong), (*n* = 9). The R packages used in this analysis included “*DescTools*” for the Kendall’s coefficient [43] and “*ggplot2*” for the graphs [44].

## Results

### Important livestock species based on difficulty of management of its diseases

The most important livestock species kept based on difficulty of management of diseases in decreasing order of rank were camels, cattle and goats with median ranks of 4, 3, and 1, respectively (*p* < 0.05). Sheep and donkeys were not considered as important on disease ranking (median rank = 0). There was no statistically significant difference in the species ranking by location (*p* > 0.05). Given that camels were ranked as most important in all study locations, participants proceeded with ranking the most important camel diseases with Hemorrhagic Septicemia (HS) and trypanosomiasis (median rank = 4 and 3, respectively) ranked top while camel flu, caseous lymphadenitis and camel pox were ranked least, (median rank = 1, 0, 0, respectively). The rankings differed significantly between diseases (*p* < 0.05) but not for study sites (*p* > 0.05).

###  Reporting of livestock diseases

### Utilization of stakeholders for disease reporting

Livestock owners were the main recipients of disease reports (median rank = 9), followed by friends and traditional healers (median rank = 7) and the Elders’ council and Private AHWs (median rank = 6), while CDRs (median rank = 2), the Chief, government AHWs and radio (median rank = 3) were the lowest ranked on receiving disease reports (Fig. 2). The communities noted that they report disease events to radios with the hope that this information can reach government AHWs. The median ranks did not significantly differ by location (*p* > 0.05) but significantly differed by stakeholder (*p* < 0.05). There was a moderate agreement amongst the stakeholders on whom the various disease events are reported to, with the livestock owner, agrovet operators, elders’ council and friends respectively, notably ranking highly for all the disease events (Table 3).

**Table 3:** A summary of utilization of stakeholders for disease reporting stratified by disease

Disease	Elders’ council	Friends	Chief	Livestock Owner	Agrovet	Traditional Healer	Radio	CDR	Elected politician (Ward level)	Govt. AHW	Kendall’s coefficient (W) and *P* value (*P*)
Haemorrhagic Septicaemia	• • • • • • • 7 (2–15)	• • • • • 5 (0–9)	• 1 (0–5)	• • • • • • • • • • • • 12 (5–24)	• • • • • • • • 8 (0–18)	• • • 3 (0–7)	• • • • 4 (0–14)	0 (0–6)	0 (0–6)	• • • 3 (0–17)	0.453**
Camel Pox	• • • • • • • 7 (0–17)	• • • • 3 (0–13)	0 (0–5)	• • • • • • • • • • • • • • 14 (4–27)	• • • • • 5 (0–21)	• • • • 4 (0–13)	0 (0–7)	0 (0–8)	• • 2 (0–7)	• • • • • 5 (0–14)	0.333*
Camel Trypanosomosis	• • • • • • • 7 (0–19)	• • • 3 (0–12)	0 (0–3)	• • • • • • • • • • • • • • • 15 (4–35)	• • • • • • • • 8 (0–13)	2 (0–11)	0 (0–6)	0 (0–10)	0 (0–6)	• • • • 4 (0–8)	0.499**
Camel Flu	• • • • • • 6 (0–17)	• • • • • • • 7 (0–14)	0 (0–4)	• • • • • • • • • • • • • • 14 (5–36)	• • • • • 5 (0–14)	• • • • 4 (0–23)	0 (0–7)	0 (0–5)	0 (0–6)	• • 2 (0–13)	0.420**
Caseous Lymphadenitis	• • • • • 5 (0–17)	• • • • 4 (0–20)	0 (0–3)	• • • • • • • • • • • • • 13 (4–34)	• • • • • • 6 (0–16)	0 (0–12)	• • • 3 (0–10)	0 (0–5)	0 (0–7)	• • • 3 (0–13)	0.365*

The Elder’s council, political leaders at the ward level, friends and Agrovet operators were the most accessible on phone based on the median rank, however the level of agreement in ranks for this factor was significantly low (Table 4). The pastoralist groups moderately agreed regarding stakeholders receiving disease reports because they were easily accessible due to their proximity to the community, while friends, livestock owners, elders’ council and politicians at ward level, respectively were ranked highly on this criterion. The pastoralists observed that elders’ council, friends and government AHWs understood them most when they reported to them a disease event and there was moderate level of agreement for ranks on this criterion. Sympathy was a major consideration for whom disease reports would be made as shown by the high level of agreement on stakeholders who ranked friends, the elders’ council and political leader at ward level, respectively (Table 4). Furthermore, there was a strong agreement among pastoralists that considerations on knowledge and expertise in utilization of synthetic medicine also influenced where disease reports would be sent (Table 4). Ranking highly on this criterion were the government AHWs and Agrovet operators, respectively. Lastly, there was a strong level of agreement among the groups concerning the stakeholders they could report to because of their ability to use their own resources to facilitate them to report further or share the disease information. Stakeholders that ranked highly for this were the elders’ council, friends and the livestock owner.

**Table 4:** Criteria influencing stakeholders to whom livestock diseases are reported to in Laisamis Sub County, Kenya

Reasons	Elders’ council	Friends	Chief	Owner	Agrovet	T. Healer	Radio	CDR	Elected politician (Ward level)	Govt. AHW	Kendall’s coefficient (W) and *P* value (*P*)
Sympathetic to one’s situation	• • • • • • • • • 9 (3–18)	• • • • • • • • • • • • 12 (6–27)	• 1 (0–3)	• • • • • • • • • • 10 (7–20)	• 1 (0–5)	• • 2 (0–8)	0 0	0 (0–6)	• • • • • • 6 (0–13)	• • 2 (0–6)	W = 0.736**
Easily accessible because of proximity to community	• • • • • • • • • • 10 (7–19)	• • • • • • • 7 (0–14)	• • • • • • 6 (0–13)	• • • • • • • 7 (1–25)	0 (0–11)	• • • • 4 (0–8)	0 (0–5)	0 (0–9)	• • • • • • 6 (0–10)	0 (0–4)	W = 0.478**
Easily accessible on phone	• • • • • • • • • 9 (0–14)	• • • • • • • 7 (2–13)	• • • 3 (0–14)	• • • • 4 (0–21)	• • • • • • 6 (0–8)	0 (0–8)	• • • • 4 (0–14)	0 (0–8)	• • • • • • • 7 (0–12)	0 (0–6)	W = 0.268**
More Knowledge and expertise in synthetic medicine and its utilization	0 (0–8)	• • • • 4 (0–8)	0 (0–3)	• • • • • 5 (0–8)	• • • • • • • • • • • • • • 14 (8–29)	0 (0–4)	• • • 3 (0–21)	0 (0–6)	0 (0–2)	• • • • • • • • • • • • • • • 15 (0–25)	W = 0.617**
Can spread information wide	• • • • • • • • • • 10 (5–26)	2 (0–8)	0 (0–13)	• • • • • • • • • 9 (0–17)	0 (0–5)	0 (0–5)	• • • • • • • • • • • • • • • 15 (2–19)	0 (0–12)	• • • • • 5 (0–13)	0 (0–5)	W = 0.524**
Understands you when you explain to them	• • • • • • • • • • • • 12 (3–15)	• • • • • • • 7 (0–10)	• • 2 (0–6)	• • • • • • 6 (1–16)	• • • • • 5 (0–10)	• • • • • 5 (0–17)	• • 2 (0–6)	0 (0–5)	• • • • 4 (0–14)	• • • • • • 6 (0–16)	W = 0.343**
Can support with own resources to facilitate reporting e.g. giving you airtime for you to call	• • • • • • • • • • • 11 (4–21)	• • • • • • • • • • • 11 (5–19)	• • 2 (0–4)	• • • • • • • • • 9 (4–18)	• • • • • • 6 (0–11)	• • • • 4 (0–7)	0 (0–7)	0 (0–4)	• • • • • • 6 (0–12)	0 (0–5)	W = 0.591**

• median counts, range in brackets, * *p* value<0.05, * *p* value <0.05, ** *p* value <0.0005, W < or = 0.3 (Weak), W > 0.3 < 0.5 (moderate),W > 0.5 (strong), (n-9)

**Fig. 3:**
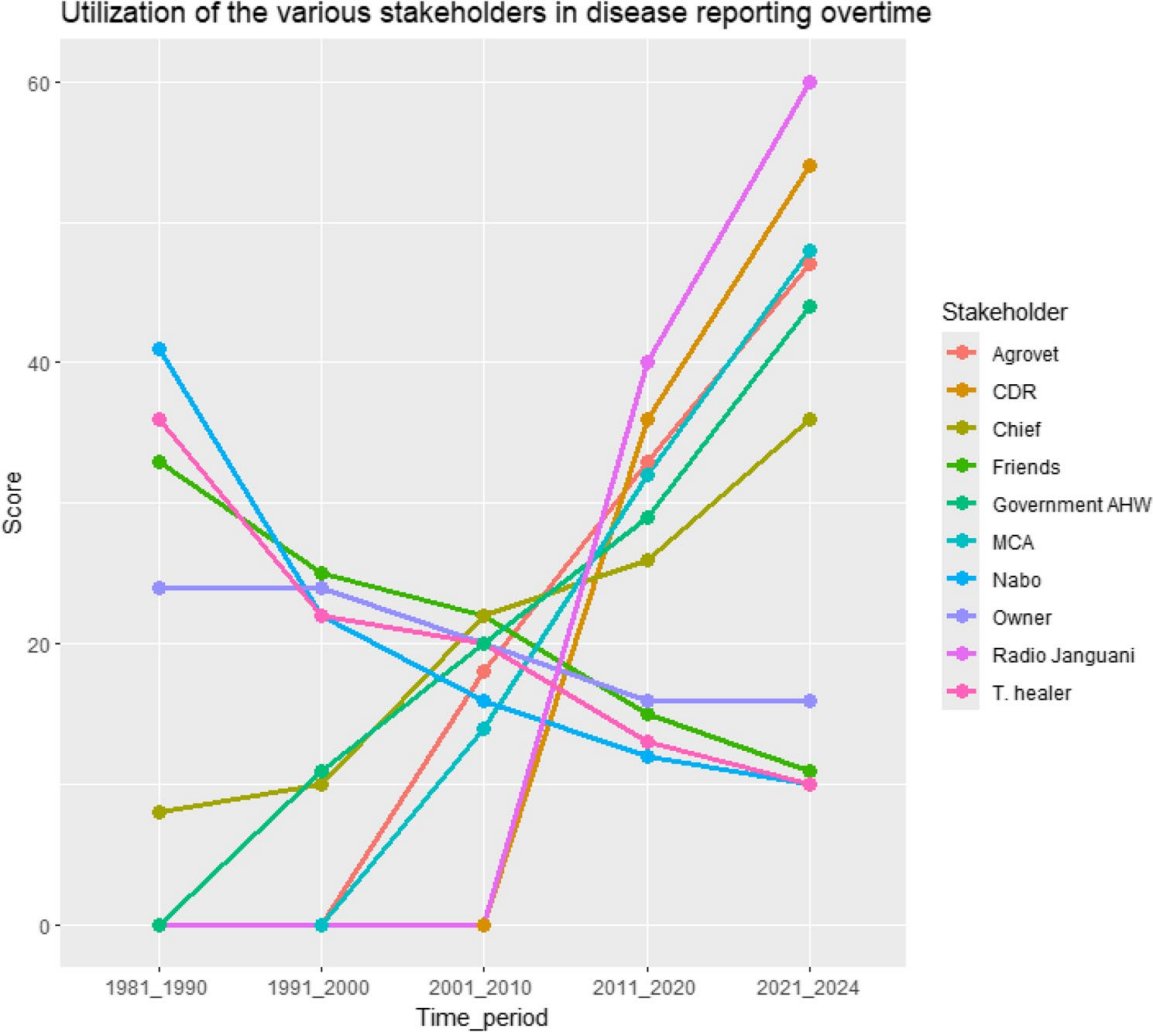
Utilization of the different stakeholders for disease reporting overtime in Laisamis Sub County, Kenya

A gradual downward trend was observed in the utilization of the elders’ council, traditional healers, friends and livestock owner, for disease reporting between 1981 and 2024 while a gradual upward trend was observed in the utilization of the chief and government AHWs during the same period. The groups highlighted that agrovets and the elected political leaders at ward level began being operational in their regions between 1991 and 2000 and the radio and CDRs in 2001–2010 and have both displayed a steep upward trend in their utilization from then, to 2024 (Fig. 3).

When the reporting methods were compared by pairwise ranking, the use of mobile phones and walking were the two most commonly used modes for disease reporting (median rank = 4 and 3, respectively), while the use of radio and motorbikes were ranked the same (median rank = 2). Motor vehicles were a mode not commonly used for disease reporting purposes in this study area. The ranking for the reporting methods did not differ by location (*p* > 0.05) but they differed by reporting method (*p* < 0.05).

When the reporting methods were stratified by disease, there was a strong agreement among the pastoralists’ groups regarding the methods used for reporting all diseases. Notably, mobile phones had a high median rank, followed by walking and lastly the use of motorbikes. The use of radios to report specific camel diseases was negligible while motor vehicles were not ranked at all (Table 5).

**Table 5:** Summary of utilization of reporting methods as stratified by disease in Laisamis Sub County, Kenya

Disease	Walking	Motorbike	Motor vehicle	Radio	Phone	Kendall’s coefficient (W) and *P* value (*P*)
Haemorrhagic Septicaemia	• • • • • • • • • • 10 (5–12)	• • • • • • • 7 (2–7)	0 (0–3)	• • 2 (0–5)	• • • • • • • • 8 (6–13)	0.685**
Camel Pox	• • • • • • • • • • 10 (5–12)	• • • • • 5 (4–8)	0 (0–3)	• 1 (0–3)	• • • • • • • • • 9 (4–13)	0.861**
Camel Trypanosomosis	• • • • • • • 7 (5–16)	• • • • • 5 (4–9)	0 (0–2)	• 1 (0–7)	• • • • • • • • 8 (4–12)	0.716**
Camel Flu	• • • • • • • 7 (3–14)	• • • • • 5 (3–9)	0 (0–2)	0 (0–6)	• • • • • • • • • • 10 (4–12)	0.681**
Caseous Lymphadenitis	• • • • • • • • 8 (3–17)	• • • • • 5 (0–10)	0 (0–2)	• 1 (0–3)	• • • • • • • • 8 (0–19)	0.702**

There was a low level of agreement for the reporting method that could be shared while reporting, with the radio, motor vehicle and mobile phone ranking highly respectively. The groups moderately agreed regarding the reporting method that could spread information faster, with the radio, motor vehicle and phone ranking highest on this criterion respectively. The pastoralists strongly agreed regarding the reporting methods that are affordable, spread information wide, easily accessible and those within the pastoralists’ control. Notably, the radio ranked high on all these criteria (Table 6).

**Table 6:** Criteria for preference of disease reporting methods by pastoralists in Laisamis Sub-county, Kenya

Criteria	Walking	Motorbike	Motor vehicle	Radio	Phone	Kendall’s coefficient (W) and *P* value (*P*)
Affordable	• • • • • • • • • • • • • • • • • • • 19 (16–29)	0 (0–6)	• • • • 4 (0–13)	• • • • • • • • • • • • • • • 15 (11–24)	• • • • • • 6 (0–10)	W = 0.809**
Fast spread of information (it can spread the information faster)	• • • • 4 (0–30)	• • 2 (0–12)	• • • • • • • • • • • • • • 14 (0–17)	• • • • • • • • • • • • • • • • • • 18 (0–25)	• • • • • • • • • • • • 12 (5–21)	W = 0.481**
Can be shared (with neighbors when we are reporting)	• • • • 4 (0–30)	• • • • • 5 (0–18)	• • • • • • • • • • • • • • 14 (0–17)	• • • • • • • • • • • • • • • • • • 18 (0–25)	• • • • • • • • 8 (0–21)	W = 0.232
Wide spread information (spreads information wider)	0 (0–17)	• • • • 4 (0–8)	• • • • • • • • • • • • • • • • • • • • • • 22 (15–28)	• • • • • • • • • • • • • • • • • • 18 (15–24)	• • • • 4 (0–10)	W = 0.755**
Easily accessible/available	• • • • • • • • • • • • • • • • • • • • 20 (12–25)	0 (0–5)	• • • 3 (0–11)	• • • • • • • • • • • • • • • • • • 18 (11–30)	• • • • • • 6 (0–18)	W = 0.783**
Method is within the livestock keeper’s control	• • • • • • • • • • • • • • • • • • • • • • 22 (9–33)	0 (0–5)	0 (0–2)	• • • • • • • • • • • • • • • • • • • 19 (7–25)	• • • • • • • • • • 10 (2–16)	W = 0.871**

• median counts, range in brackets, * *p* value > 0.05, * *p* value <0.05, ** *p* value < 0.0005, W < or = 0.3 (Weak) W > 0.3 < 0.5 (Moderate), W > 0.5 (strong), (n=9)

**Fig. 4:**
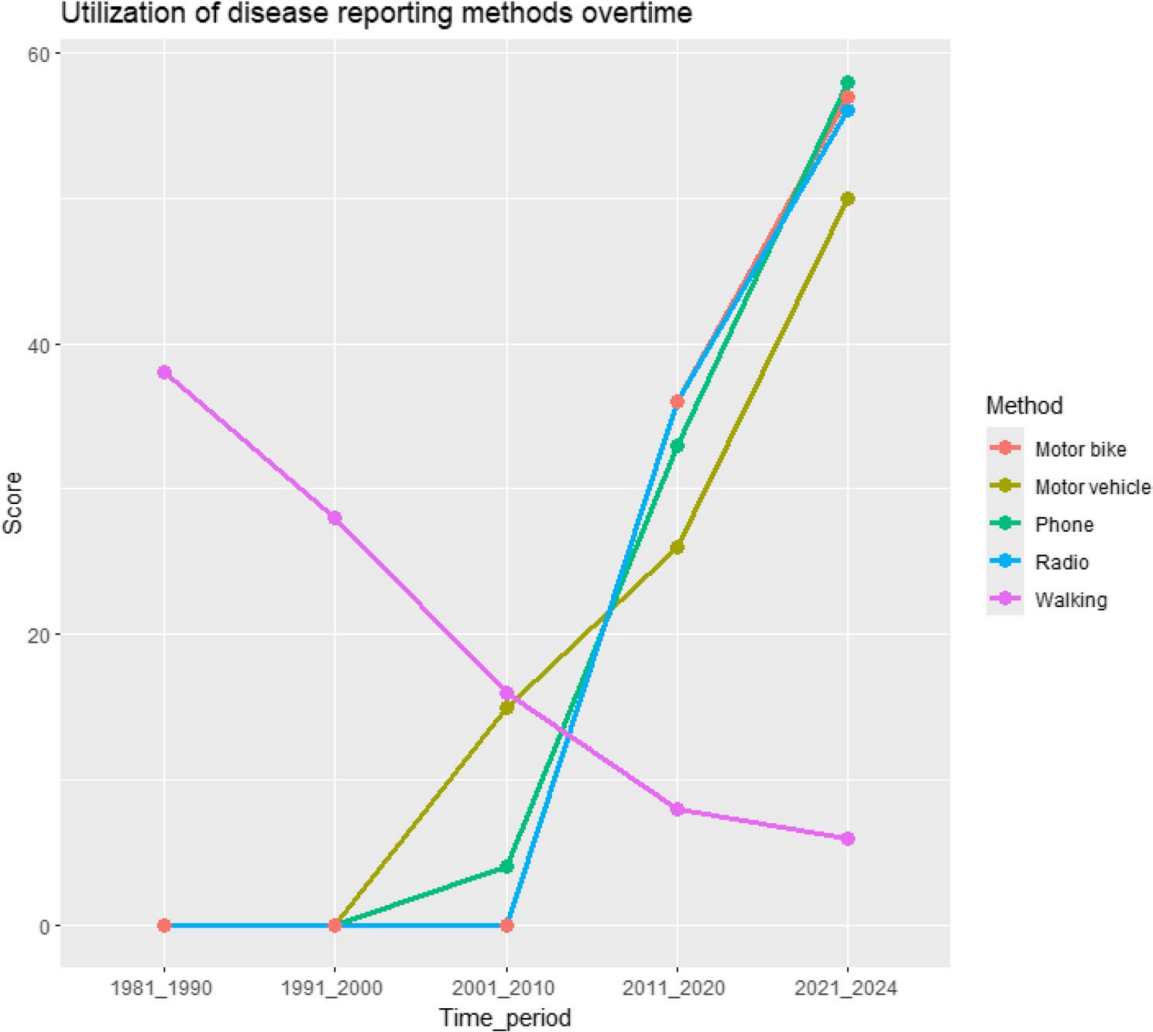
Utilization of reporting methods overtime in Laisamis Sub County, Kenya

Over the years, it was observed that walking, as a method used to report disease events became less popular and motor vehicles and phones started being utilized for reporting livestock diseases between 1981 and 2000, while motor bikes, between 2001 and 2010. All methods show a gradual upward trend in their utilization from then to 2024 apart from walking (Fig. 4). The communities clarified that as much as radios and radio stations may have existed before 2001, the stations available were in languages they could not understand. Therefore, the highlighted time interval is representative of when radio stations like Radio *Janguani* that air programs in native languages were established.

### Response to livestock disease events

**Fig. 5:**
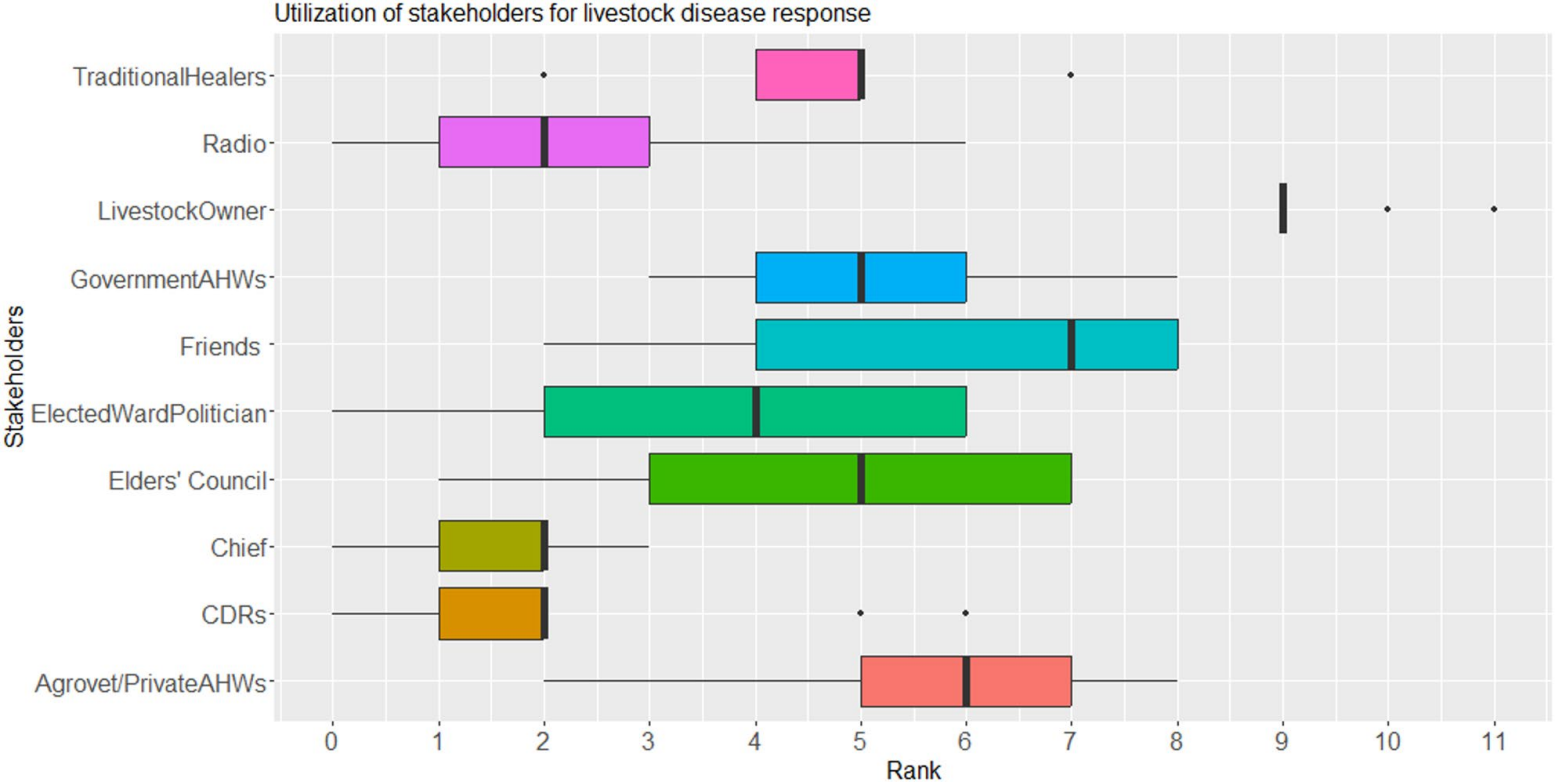
Rankings for stakeholders as utilized for livestock disease response in Laisamis sub county, Kenya

In most cases, the livestock owner responded to disease events in their herds (median rank = 9). Other common response mechanisms involved contacting their friends who were pastoralists as well (median rank = 7) and agrovet operators/private practitioners (median rank = 6) (Fig. 5). The rankings did not differ by location (*p* > 0.05) but they did by stakeholder (*p* < 0.05). When stratified by disease occurrences, the pastoralist groups moderately agreed concerning the stakeholders that responded to all the five priority diseases. Generally, the livestock owner was ranked high as important for responding to all disease events, followed by the Agrovet operators, Elders’ council and friends. The traditional healers were ranked only for suspected cases of Haemorrhagic septicaemia and Camel pox while the government AHW was ranked highest for suspected cases of Camel Trypanosomosis and Caseous Lymphadenitis (Table 7).

**Table 7:** Utilization of livestock disease response stakeholders as stratified by disease in Laisamis Sub County, Kenya

Disease	Elders’ council	Friends	Chief	Livestock Owner	Agrovet	Traditional Healer	Radio	CDR	Elected politician (ward level)	Govt. AHW	Kendall’s coefficient (W) and *P* value (*P*)
Haemorrhagic Septicaemia	• • • • • • • • • • 10 (0–15)	• • • • • • • • 8 (0–18)	0 (0–6)	• • • • • • • • • • • 11 (5–25)	• • • • • • • 7 (0–15)	• • • • 4 (0–15)	0 (0–4)	0 (0–6)	0 (0–9)	• • • 3 (0–13)	0.404**
Camel pox	• • • • • 5 (0–23)	• • • • 4 (0–15)	0 (0–6)	• • • • • • • • • • • 11 (4–35)	• • • • • • • 7 (0–14)	• • • • 4 (0–22)	0 (0–4)	0 (0–7)	• • 2 (0–8)	• • • • 4 (0–15)	0.310*
Camel Trypanosomosis	• • • 3 (0–14)	• • • • • • 6 (1–14)	0 (0–5)	• • • • • • • • • • • • • • 12 (5–36)	• • • • • • • • 8 (0–27)	0 (0–11)	0 (0–4)	0 (0–7)	0 (0–7)	• • • • • • • 7 (0–17)	0.467**
Camel Flu	• • • • • • • 7 (0–20)	• • • • 4 (0–11)	0 (0–6)	• • • • • • • • • • • • • 13 (4–34)	• • • • • • • 7 (0–17)	0 (0–14)	0 (0–10)	0 (0–5)	0 (0–7)	0 (0–21)	0.379**
Caseous Lymphadenitis	• • • • 4 (0–16)	• • • 3 (0–15)	0 (0–2)	• • • • • • • • • • • • • • • • • • • • • 21 (2–30)	• • • • • • • • • 9 (0–23)	0 (0–21)	0 (0–2)	0 (0–7)	0 (0–8)	• • • • • • • 7 (0–15)	0.413**

The pastoralists strongly agreed regarding the stakeholders that were knowledgeable in indigenous methods of managing diseases where the traditional healer was ranked highest followed by the Elders’ council, friends and lastly the livestock owner. The pastoralists also strongly agreed on the stakeholders that offer quick response with the livestock owner, friends, elders’ council and the traditional healers ranked highly for this. There was also strong agreement for the stakeholders considered to offer services on credit, with the Elders’ council, friends and agrovet ranking high. The pastoralist groups moderately agreed on the stakeholder that could support with their own resources to facilitate response where the ward level politician ranked highest (Table 8).

**Table 8:** Criteria for preference of stakeholders involved in livestock disease response in Laisamis Sub County, Kenya

Criteria	Elders’ council	Friends	Chief	Livestock Owner	Agrovet	Traditional Healer	Radio	CDR	Elected politician (ward level)	Govt. AHW	Kendall’s coefficient (W) and *P* value (*P*)
Provides quicker response	• • • • • • • • • 9 (6–20)	• • • • • • • • • • 10 (6–25)	0 (0–2)	• • • • • • • • • • • • • 13 (4–18)	0 (0–15)	• • • • • • • 7 (0–12)	0 (0–9)	0 (0–3)	0 (0–10)	0 0	W = 0.661**
Has special indigenous knowledge (e.g. fracture management, assisted delivery)	• • • • • • • • • • • 11 (0–16)	• • • • • • • • • • • 11 (6–15)	0 (0–4)	• • • • • • • • • • 10 (6–19)	0 (0–4)	• • • • • • • • • • • • • • 14 (0–32)	0 (0–6)	0 (0–5)	0 (0–2)	0 (0–0)	W = 0.802**
Can consult others if they do not know the remedy	• • • • • • • • • • 10 (4–25)	• • • • • • • • 8 (4–20)	0 (0–5)	• • • • • • • • • • • • 12 (4–20)	0 (0–6)	• • • • 4 (0–9)	0 (0–10)	0 (0–8)	• • • • • 5 (0–15)	0 (0–0)	W = 0.454**
Can offer services on credit	• • • • • • • • • • • 11 (0–25)	• • • • • • • • • • • 11 (5–29)	0 (0–0)	• • • • • • • 7 (0–14)	• • • • • • • 7 (0–27)	• • • • 4 (0–13)	0 (0–0)	0 (0–3)	0 (0–11)	0 (0–6)	W = 0.609**
Can support with own resources to facilitate response e.g. giving an animal to sell to buy drugs	• • • • • • • • 8 (0–24)	• • • • • • • • 8 (3–22)	0 (0–3)	• • • • • • • 7 (0–28)	0 (0–10)	• • • • • 5 (0–12)	0 (0–0)	0 (0–5)	• • • • • • • • • 9 (0–13)	0 (0–14)	W = 0.449**

**Fig. 6:**
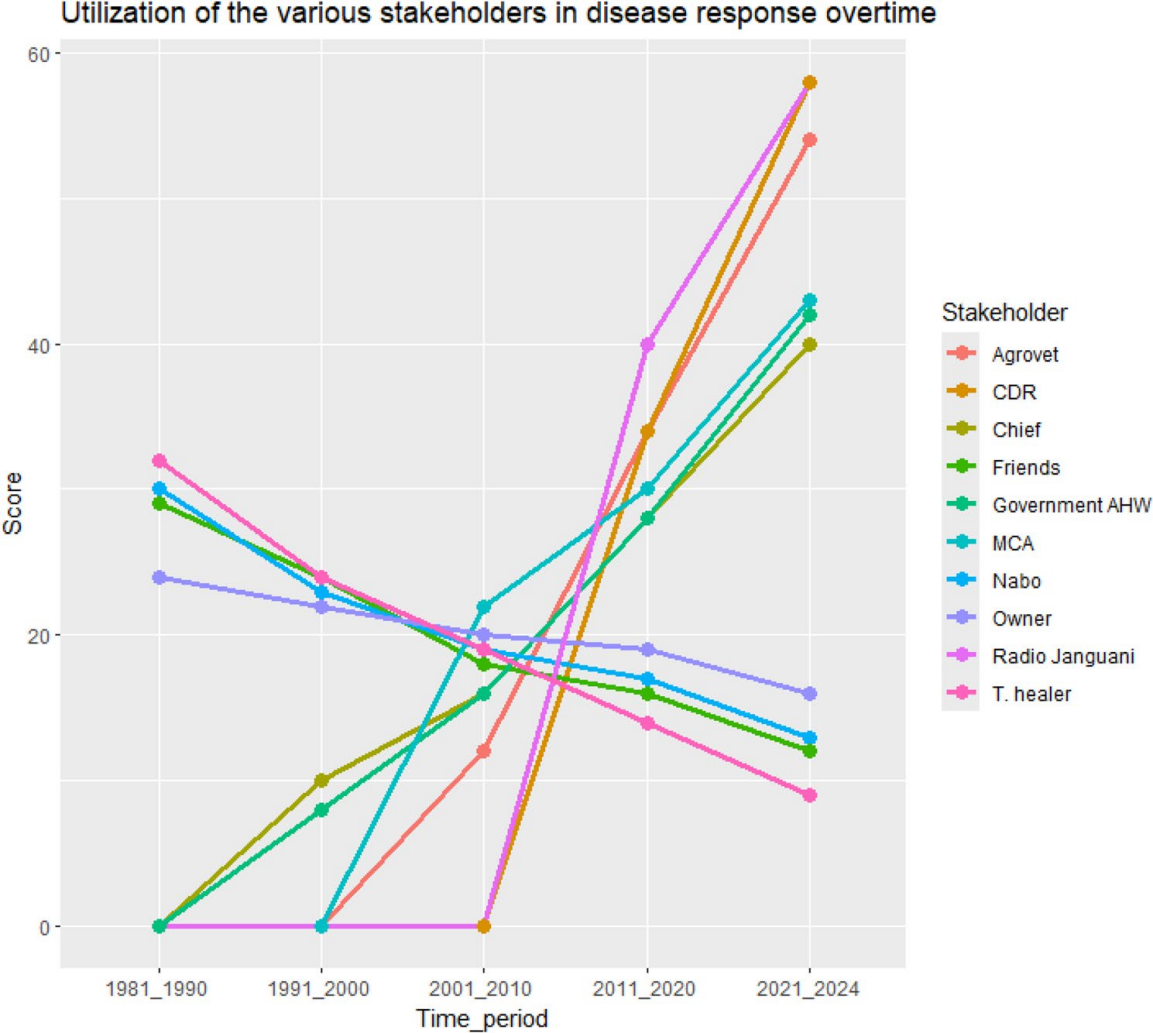
Utilization of the various stakeholders in disease response overtime in Laisamis Sub County, Kenya

A gradual downward trend was observed in the utilization of the traditional healers, friends and livestock owner, for disease response between 1981 and 2024 while a gradual upward trend was observed in the utilization of the chief and government AHWs within the same period (Fig. 6). The groups highlighted that agrovets and the ward level political leaders began being operational in their regions between 1991 and 2000, and the radio and CDRs in 2001–2010, they have all displayed a steep upward trend in their utilization from then, to 2024 (Fig. 6).

**Fig. 7:**
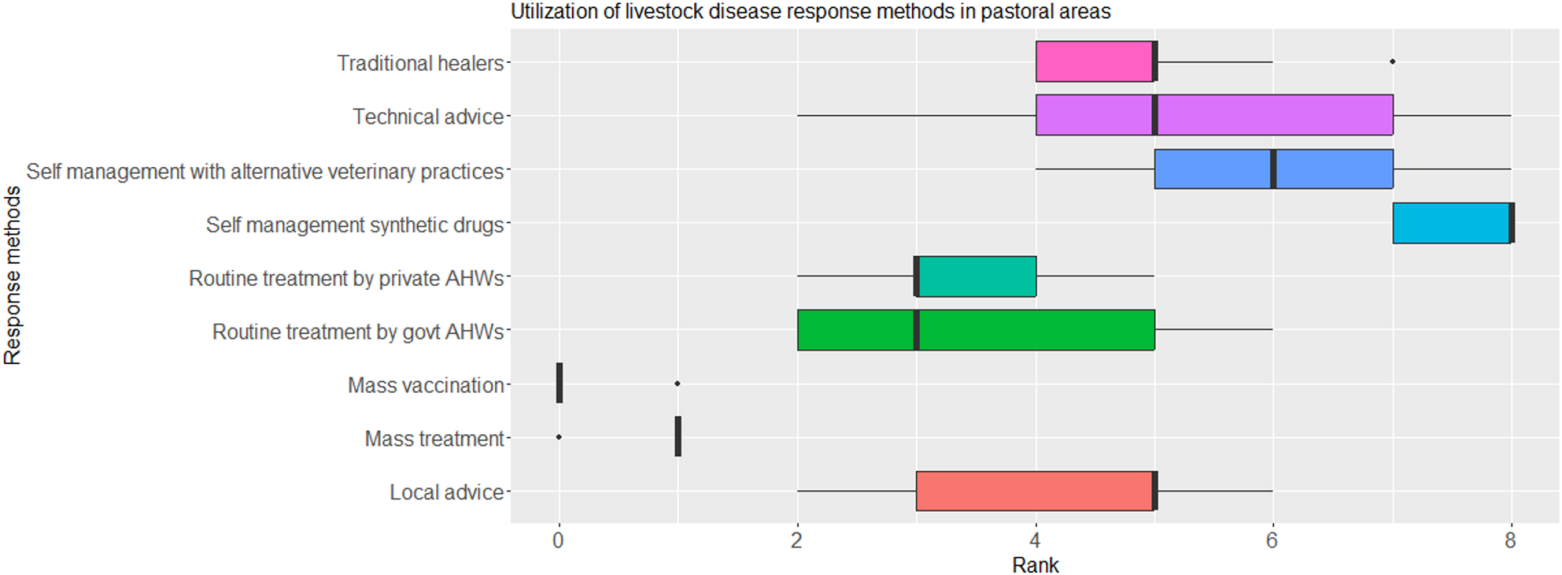
Rankings for livestock disease response methods as utilized by pastoralists in Laisamis Sub County, Kenya

Pastoralists mostly managed livestock diseases by themselves through the use of synthetic drugs and alternative veterinary practices (medians = 8 and 6 respectively). The pastoralists highlighted that Traditional healers were also knowledgeable about synthetic drugs and could be consulted for advice on the best drug of use for a given disease event, but also prescribed synthetic drugs incase their interventions failed. The pastoralists also noted that as much as they owned syringes and had access to drugs, they were not confident of which drugs to use when, or their administration. Mass treatment and mass vaccination were the least utilized methods for disease response (medians = 1 and 0 respectively). The median rank did not significantly differ by location (*p* > 0.05) but significantly differed by response method (p < 0.05) (Figure 7).

Generally, there was moderate agreement on the utilization of the different response methods for the priority diseases. Based on the ranks, pastoralists majorly managed all diseases by themselves using synthetic drugs, alternative veterinary practices and through traditional healers in decreasing order. Government AHWs routinely managed mostly haemorrhagic septicaemia and camel pox as reflected by the high ranks (Table 9).

**Table 9:** Utilization of response methods by disease in Laisamis Sub County, Kenya

Disease	Mass treatment	Mass vaccination	Local advice	Technical advice	Routine - private AHWs	Routine - government AHWs	Self – synthetic	Self – alternative	Traditional healers	Kendall’s coefficient (W) and *P* value (*P*)
Haemorrhagic Septicaemia	0 (0–2)	• • 2 (0–11)	• • 2 (0–15)	0 (0–3)	• • 2 (0–15)	• • • • • • • • 8 (0–12)	• • • • • • • 7 (0–29)	• • • • • • • • 8 (0–21)	• • • • 4 (0–12)	0.330*
Camel Pox	0 (0–13)	0 (0–11)	0 (0–9)	• • 2 (0–5)	0 (0–12)	• • • • • • 6 (0–12)	• • • • • • • • • • • 11 (0–17)	• • • • • • • • • • 10 (0–27)	• • • • • • • 7 (0–12)	0.349*
Camel Trypanosomosis	0 (0–11)	0 (0–7)	• • • • • • 6 (0–12)	0 (0–7)	0 (0–12)	0 (0–12)	• • • • • • • • • • • • • • • 15 (0–28)	0 (0–27)	• • • • • • • 7 (2–33)	0.364**
Camel Flu	0 (0–13)	0 (0–11)	• • • 3 (0–14)	0 (0–7)	0 (0–5)	0 (0–11)	• • • • • • • • • • • • 12 (0–28)	• • • • • 5 (0–14)	• • • • • • • 7 (0–31)	0.336*
Caseous Lymphadenitis	0 (0–10)	0 (0–11)	0 (0–7)	• • 2 (0–7)	• • 2 (0–18)	0 (0–17)	• • • • • • • • • • • • • • • • 16 (0–22)	• • • • • • • • • 9 (0–18)	0 (0–12)	0.264*

• median counts, range in brackets, * *p* value > 0.05, * *p* value <0.05, ** *p* value < 0.0005, W < or = 0.3 (Weak) W > 0.3 < 0.5 (Moderate), W > 0.5 (strong), (n=9)

The methods for disease response were chosen based on their integration of indigenous knowledge, involvement of a technician, number of animals they can manage, how quick they can offer response and affordability (Table 10). The pastoralist groups strongly agreed on the response methods they used because they were based on indigenous methods, the response methods that were used because a technician was involved and the methods used because they were affordable. There was moderate agreement on the methods that were used because they provided quicker response, ranked notably high among these was treatment using alternative veterinary methods, treatment using synthetic drugs and treatment by agrovets/private AHWs. There was moderate agreement about the response methods that are preferred because through them many herds were covered (Table 10).

**Table 10:** Criteria for preference of different disease response methods in Laisamis Sub County, Kenya

Criteria	Mass treatment	Mass vaccination	Local advice	Technical advice	Routine - private AHWs	Routine - governmentt AHWs	Self – synthetic	Self – alternative	Traditional healers	Kendall’s coefficient (W) and *P* value (*P*)
Affordable	• • • • 4 (0–8)	• • • 3 (0–9)	• • • • • 5 (3–10)	• • • • • • 6 (0–10)	• • 2 (0–6)	• • 2 (0–4)	• • • • • • 6 (0–16)	• • • • • • • • • • 10 (5–16)	• • • • • • • 7 (3–11)	W = 0.516 **
The method covers many herds (benefits many livestock keepers)	• • • • • • • • • • • • • • • 15 (0–16)	• • • • • • • • • • • 11 (0–17)	• 1 (0–6)	• • • • 4 (0–7)	0 (0–10)	0 (0–11)	• • • • • 5 (0–15)	• • • • • • • • 8 (0–15)	0 (0–9)	W = 0.359**
Quicker response	0 (0–30)	0 (0–15)	• • 2 (0–8)	• • 2 (0–8)	• • • • • • 6 (0–11)	0 (0–3)	• • • • • • • • • 9 (0–24)	• • • • • • • • • • • • 12 (0–22)	• • • • • • • 7 (0–12)	W = 0.466**
Based on indigenous knowledge (local remedies)	0 (0–0)	0 (0–0)	• • • • • • • • • • • • 12 (7–16)	0 (0–3)	0 (0–0)	0 (0–3)	0 (0–9)	• • • • • • • • • • • • • • • 15 (7–20)	• • • • • • • • • • • • • • • 15 (9–30)	W = 0.885**
Needs the knowledge of a technician	• • • • 4 (0–9)	• • • • • 5 (0–18)	0 (0–27)	• • • • • • • • 8 (0–16)	• • • • • • • 7 (0–16)	• • • • • • • • 8 (0–13)	• • • • • • • • 8 (0–18)	0 (0–1)	0 (0–3)	W = 0.573 **

• median counts, range in brackets, * *p* value > 0.05, * *p* value <0.05, ** *p* value < 0.0005, W < or = 0.3 (Weak), W > 0.3 < 0.5 (Moderate), W > 0.5 (strong), (n=9)

**Fig. 8:**
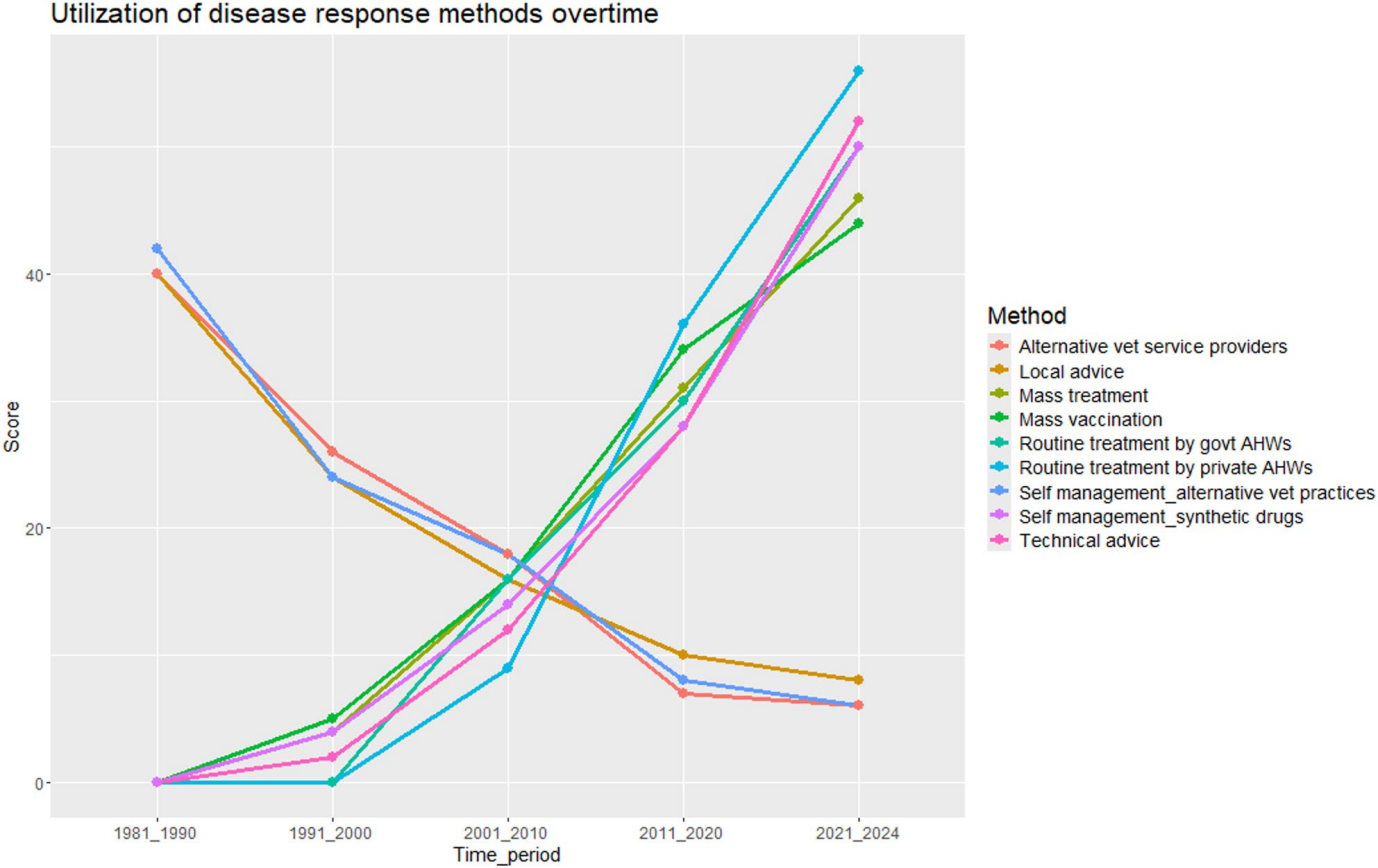
Utilization of the various disease response methods overtime

Pastoralists treating animals by themselves using synthetic drugs, technical advice given to manage disease events and mass treatment are response methods that have been used from 1981 to 2024, and have experienced a gradual increase overtime (Fig. 8). The increased penetration of phones in pastoral areas was mentioned as an augmenting factor to self-management of livestock diseases by pastoralists as most times the solution is just a call away. Government and private AHWs started routinely visiting pastoralists for response interventions beginning in the 1990s and this has gradually increased from then to 2024. The pastoralists noted that before the 1990s technical AHWs were present but they were very low in numbers, they treated specific species, especially cattle and they only lived in towns. On the other hand, the use of alternative veterinary medicine, local advice given to manage disease response and use of traditional healers are methods that have been used even before the 1980s, however, their utilization has gradually reduced overtime. Reasons given by the communities for the reduction in utilization of traditional approaches included the fact that they are trial and error and lack of regulation leading to a lot of quacks in the practice (Fig. 8).

## Discussion

This study used participatory epidemiology to describe the passive surveillance system in pastoral settings, alongside important criteria/considerations for utilization and preference for reporting and response stakeholders and methods by pastoralists. Results showed that amongst other species, camels are important with diseases that are difficult to manage. Overall, decision and motivation for choice of disease reporting modalities and recipients of disease reports were influenced by similar factors. Most prominent were empathy and perceived technical knowledge of the recipient, ability to integrate indigenous knowledge and synthetic drugs as well as affordability. For certain diseases like hemorrhagic septicemia, reporting avenues with a wider reach/audience and shorter communication time were preferred.

The most important camel diseases were Haemorrhagic Septicaemia (HS), camel pox, camel trypanosomosis, camel flu and caseous lymphadenitis. The important diseases mentioned are consistent with those highlighted in Kerio, Central Turkana by Bett et al. [11] and by Lelenguyah et al. [45] in Samburu county in Kenya. HS and camel trypanosomosis were among the diseases of importance in camels in a study by Mochabo et al. [46] in Turkana. The endemic status of these diseases in the region has probably been maintained by complex combinations of risk factors. Besides marginalization, studies documenting risk factors for these particular diseases have noted; long distances from the agrovets where drugs can promptly be purchased, harsh weather conditions comprising of frequent droughts interspersed with irregular periods of rainfall, geographical constraints, inadequate veterinary services, drug resistance, low primary caregiver/pastoralist knowledge on the right veterinary drugs to use, disease etiologies and prevention, among others contribute to the endemicity and spread of disease in ASAL areas [47, 48].

Livestock disease reporting is particularly challenging in resource scarce settings where disease events often go unreported. The criteria highlighted by the pastoralists in this study mirror pastoralists’ motivations in reporting diseases. Some of the important criteria for pastoralists for stakeholders to whom they report disease events to were accessibility either due to proximity to the community or on phone. Government AHWs scored really low on these, similar to observations in Bolivia where accessibility of the Official Veterinary Services and their offices’ distances from the community were factors that enabled under reporting [31]. Because of these, community members believed that the veterinarians were disinterested in their problems and were not motivated to help [31]. This finding is also consistent with [49, 50] that found that distance of the veterinarians from the community influenced the reporting behavior of pastoralists in Geraldton in Western Australia. Notwithstanding, inference can be made from a scoping review by Gates et al. [51], that community livestock disease reports can be improved by authorities and animal health institutions building good relationships and trust with the community, clarifying and simplifying reporting procedures, making reporting mandatory through the law and fortification of these through acceptable enforcement and provision of incentives for reporting where possible. Phones were the most preferred method for reporting livestock diseases with their utilization increasing to 2024. This is substantiated by the trove of evidence showing increased penetration and use of phones and ICT in disease reporting in Africa [52–57].

Agrovets/private AHWs were the most preferred stakeholders for disease response after the pastoralists themselves, with their utilization increasing overtime. They also scored high on the criterion of ability to offer services on credit. This is consistent with several findings. Among other studies, in Kakamega, Kenya, private AHWs ranked highest with regard to accessibility, attitude, access to information and service provision on credit [58], in Uganda, private AHWs in the study sites had a high average rank, the community especially appreciated that they had a healthy working relationship with paraprofessionals, they owned drug shops and charged lower fees than government AHWs [59], while in Ethiopia livestock keepers were most satisfied with and scored private AHWs highest across all production systems and for all socioeconomic groups except farmers with very large herds [60]. The majority of respondents in Bena-Tsemay, Ethiopia noted that they received acaricide use information from private drug shops [61]. However, the findings are in contrast with Kebede et al. [62] that observed only 20.33% of farmers preferring private AHWs in comparison to the 58.54% that preferred government AHWs.

Pastoralists also preferred to respond to livestock diseases by themselves through use of synthetic drugs. Examples of the drugs that they had are presented in [24], this should elicit further research into antimicrobial stewardship in pastoral settings [63]. This method of response ranked highly for all the diseases. This finding is consistent with a study by Mangesho et al. [64] in Tanzania where 90% of Maasai pastoralists indicated that their immediate response to a disease event would be to use drugs within their *boma* or the neighbor’s, because they believed themselves to be “experts” in animal health and Maasai men, especially, potrayed themselves as custodians of veterinary drug knowledge and its administration. In case this failed, they would then seek advice from their friends, then elders before seeking for technical personnel. The authors noted that lack of knowledge on identification of a livestock disease and its corresponding treatment could be an embarrassment. Its noteworthy that pastoralists treating by themselves using synthetic drugs has also increased overtime, this has been realized with the reduction in use of traditional healers, local advice and alternative veterinary medicine in responding to livestock diseases. Theories that explain this reduction are summarized in a review by Wanzala et al. [65] including the fact that traditional approaches are trial and error, less researched, documented, systematic and formalized. These approaches can also not keep up with the ever-changing disease landscape. However, as much as their utilization is decreasing, traditional healers still have important roles they play in animal health management in pastoral communities because of their proximity to and the trust beholden to them from the community and because of the limitations that deter technical personnel from reaching all community members. They fill an animal health service delivery void in pastoral areas, albeit not with conventional medicine. Therefore, innovative ways can be explored to harness their roles for the benefit of disease outbreak investigations and surveillance. Similar practice has been reported in human health surveillance in Uganda where important information on disease events is gathered from traditional healers for early detection and management of outbreaks [66].

Consideration and incorporation of local perspectives is important in the process of enhancement of surveillance and response systems. An 18-month trial in Tanzania adopted this approach in developing and testing a surveillance system. In the intervention districts, community members being allowed to design the system based on ease of use and sustainability, from their points of view, led to improvements in the criteria of simplicity and acceptability. Involvement of more stakeholder nodes in this trial led to substantial improvements in sensitivity and representativeness of the system [67]. In the same light, this study gathered what is considered important by pastoralists in the process of reporting and responding to livestock diseases, for the benefit of interventions aimed towards improvement of the system. This study primarily focused on pastoralists, this could be a limitation, future studies would benefit from thorough engagement of other important stakeholders that have roles in strengthening disease reporting and response including: government and private AHWs, NGOs, local and national administrators among others. Discussions with these different groups could be held in disaggregation to facilitate for more open, free, unbiased and multidimensional discourses. Secondly, the perspectives captured were from two closely related tribes, future studies could consider engaging more than one social group in varied agro ecological zones and livestock production systems for the benefit of comparative analyses. Lastly, the discussions were held with men, women too, play important roles in pastoral livestock keeping, future studies should disaggregate discussions by gender. More robust and rigorous study designs like randomized control trials focusing on local factors/criteria influencing surveillance to test the legitimacy of local perspectives and their effects on disease reporting and response, can be considered.

### Conclusions and recommendations

We observed that decisions on disease reporting among pastoralists were predominantly influenced by accessibility, proximity and affordability of available options while choice of response was mainly based on response time, technical knowledge and affordability of disease response options. Through understanding of communities’ behavioral influences and interactions, we can identify opportunities for developing adoptable, sustainable and affordable surveillance systems that are meaningful to the primary stakeholders and respond to demands of improvements in animal health. For this cause, participatory and co-creation models, among others, can be utilized for stakeholder engagement and contribution for surveillance system designs.

There has been a shift from traditional approaches of disease management towards increased use of formal actors like government animal health workers and agrovets, reflecting changes in access and trust. Despite this, our results showed mass treatment and vaccination remain underutilized, both receiving the least score on being received promptly at the time they’re needed. However, we recommend further in-depth research into possible causes of low frequencies of mass interventions from animal health authorities but also possible vaccination hesitancy on the community side. There is the need for further research into stewardship of veterinary medicines by the pastoralists and policy focus on guiding safe and informed disease management practices within community-based surveillance systems. To improve technical disease response service delivery to pastoralists, the government can consider policy and financial incentives and public – private partnership frameworks that can motivate diversified forms of private services to pastoralists, the varieties of which can include, but not limited to, contractual service delivery, subsidies or cost – sharing models.

Thus, insights gained from this study should inform the enhancement of surveillance activities in pastoral areas. Further, legislative reforms and policies to streamline data collection through pastoral communities’ reporting structures would strengthen existing disease surveillance systems in ASALs.

### Ethics statement

The Faculty of Veterinary Medicine, Biosafety, Animal Use and Ethics Committee (FVM BAUEC) of the University of Nairobi, Kenya reviewed and approved this study in accordance with the ethical standards laid down in the 1964 Declaration of Helsinki and its later recommendations, and the National Commission for Science, Technology and Innovation (NACOSTI), in Nairobi, Kenya issued research permits. Permission to conduct the research was also granted by the office of the County Directorate of Veterinary Services, Marsabit and the community leadership within the study areas. Furthermore, informed consent was obtained from all research participants by signing an informed consent form, and a locally recruited research assistant read and translated the study objectives and purpose to inform participants’ consent. The consent form also included confidentiality clauses that were read and explained to all participants.

## Supplementary Information

Supplementary Material 1.

Supplementary Material 2.

## Data Availability

The datasets and codes used in writing this article are available from the authors at reasonable request.

## Abbreviations

AHW: Animal Health Worker
ASALs: Arid and Semi–Arid Lands
CBAHWs: Community Based Animal Health Workers
NGOs: Non–Governmental Organizations
PE: Participatory Epidemiology
CDRs: Community Disease Reporters
FGDs: Focus Group Discussions
Govt.: Government
MCA: Member of County Assembly (Elected ward level political leader)
