# Supplementary material for: Pastoralists’ utilization and preferences for stakeholders and methods in livestock disease reporting and response in Northern Kenya: a participatory study
Source: BMC Vet Res. 2026 Feb 17;22:183. doi: 10.1186/s12917-026-05286-1 (PMC13015140; doi:10.1186/s12917-026-05286-1)
Supplement: Supplementary file 1 — Supplementary Material 1. [file 12917_2026_5286_MOESM1_ESM.docx]

**PATICIPATORY EPIDEMIOLOGY DATA COLLECTION TOOL**

**LIVESTOCK DISEASE REPORTING**

**IMPORTANT ANIMAL SPECIES AND ITS DISEASES**

For each group, please follow the activity flow as below:

1. Important animal species prioritized based on difficulty of management of diseases

Cattle, camels, goats, sheep or donkeys.

Use **Pairwise ranking.**

1. Diseases of most important species prioritized.

Use **Pairwise ranking.**

**LIVESTOCK DISEASE REPORTING – METHODS**

1. What is the preference for different reporting methods and why?

PE method – **Pairwise Ranking**

1. Preference for different reporting methods for these diseases and why? **(Use Matrix scoring)**

Stratify by the top 5 diseases as earlier mentioned

1. Proportional use of reporting methods overtime.

Use the local events from the different communities

Selected time frames are: 1981 - 1990, 1991 - 2000, 2001 – 2010, 2011 – 2020, 2021 – 2024

(Use **timeline** with **proportional piling**)

1. Reporting methods vs highlighted considered criteria **(Use Matrix scoring)**

**LIVESTOCK DISEASE REPORTING – STAKEHOLDERS**

1. What is the preference for different reporting stakeholders and why?

PE method – **Pairwise Ranking**

1. Preference for different reporting stakeholders for these diseases and why? **(Use Matrix scoring)**

Stratify by the top 5 diseases as earlier mentioned

1. Proportional use of reporting stakeholders overtime.

Use the local events from the different communities

Selected time frames are: 1981 - 1990, 1991 - 2000, 2001 – 2010, 2011 – 2020, 2021 – 2024

(Use **timeline** with **proportional piling**)

1. Reporting stakeholders vs considered criteria **(Use Matrix scoring)**

**LIVESTOCK DISEASE RESPONSE – METHODS**

1. What is the preference for different response methods and why?

PE method – **Pairwise Ranking**

1. Preference for different response methods for these diseases and why? **(Use Matrix scoring)**

Stratify by the top 5 diseases as earlier mentioned

1. Proportional use of response methods overtime.

Use the local events from the different communities

Selected time frames are: 1981 - 1990, 1991 - 2000, 2001 – 2010, 2011 – 2020, 2021 – 2024

(Use **timeline** with **proportional piling**)

1. Response Methods Vs the considered criteria **(Use Matrix scoring)**

**LIVESTOCK DISEASE RESPONSE – STAKEHOLDERS**

1. What is the preference for different response stakeholders and why?

PE method – **Pairwise Ranking**

1. Preference for different response stakeholders for these diseases and why? **(Use Matrix scoring)**

Stratify by the top 5 diseases as earlier mentioned

1. Proportional use of response methods overtime.

Use the local events from the different communities

Selected time frames are: 1981 - 1990, 1991 - 2000, 2001 – 2010, 2011 – 2020, 2021 – 2024

(Use **timeline** with **proportional piling**)

1. Response options vs considered criteria **(Use Matrix scoring)**

**CHECKLIST**

| **REPORTING STAKEHOLDERS** | **REPORTING METHODS** | **RESPONSE STAKEHOLDERS** | **RESPONSE METHODS** |
| --- | --- | --- | --- |
| Nabo | 1 Walking | Nabo | 1 Mass treatment |
| Friends | 2 Motor vehicle | Friends | 2 Mass vaccination |
| Chief | 3 Radio | Chief | 3 Local advice |
| Owner | 4 Phone | Owner | 4 Technical advice |
| Agrovet | 5 Motorbike | Agrovet | 5 Routine treatment by private AHWs |
| T. healer |  | T. healer | 6 Routine treatment by govt AHWs |
| Radio |  | Radio | 7 Self-management – synthetic drugs |
| CDR |  | CDR | 8 Self-management – alternative vet practices |
| MCA |  | MCA | 9 Alternative vet service providers |
| Government AHW |  | Government AHW |  |

**Criteria influencing preference of stakeholders to whom diseases are reported to**

1. Sympathetic to your situation
2. Easily accessible because of proximity to community
3. Easily accessible on phone
4. More Knowledge and expertise in synthetic medicine and its utilization (for diseases they do not know how to manage/for drugs)
5. Wide spread information (spreads information wider)
6. Understands you when you explain to them
7. Can support with own resources to facilitate reporting e.g giving you airtime for you to call

**Criteria influencing utilization of stakeholders that respond to livestock diseases**

1. Quicker response
2. With special knowledge (e.g fracture management, assisted delivery)
3. Can consult others if they do not know the remedy
4. Can offer services on credit
5. Can support with own resources to facilitate response e.g giving an animal to sell to buy drugs

**Criteria influencing utilization of reporting methods**

1. Affordable
2. Fast spread of information (it can spread the information faster)
3. Can be shared (with neighbors when we are reporting)
4. Wide spread information (spreads information wider)
5. Easily accessible/available
6. Method is within the livestock keeper’s control

**Criteria influencing utilization of response methods**

1. Affordable
2. The method covers many herds (benefits many livestock keepers)
3. Quicker response
4. Based on indigenous knowledge (local remedies)
5. Needs the knowledge of a technician
