# Supplementary material for: Pastoralists’ utilization and preferences for stakeholders and methods in livestock disease reporting and response in Northern Kenya: a participatory study
Source: BMC Vet Res. 2026 Feb 17;22:183. doi: 10.1186/s12917-026-05286-1 (PMC13015140; doi:10.1186/s12917-026-05286-1)
Supplement: Supplementary file 2 — Supplementary Material 2. [file 12917_2026_5286_MOESM2_ESM.docx]

**Supplementary Material 2**

| **Characteristic** | **Category** | **Count (n = 70)** | **Percentage** |
| --- | --- | --- | --- |
| Age | 18 – 30 | 6 | 8.6 |
|  | 31 – 40 | 13 | 18.6 |
|  | 41 – 50 | 26 | 37.1 |
|  | 51 – 60 | 17 | 24.3 |
|  | Above 60 | 8 | 11.4 |
|  |  |  |  |
| Education level | None | 49 | 70 |
|  | Primary level | 11 | 15.7 |
|  | Secondary level | 7 | 10 |
|  | Tertiary level | 3 | 4.3 |
|  |  |  |  |
| Role in community | Livestock Owner/Herder | 36 | 51.5 |
|  | Community Disease Reporter | 5 | 7.1 |
|  | Elder | 22 | 31.4 |
|  | Traditional Healer | 7 | 10 |
|  |  |  |  |
| Sublocation | Merille | 8 | 11.4 |
|  | Tirgemo | 7 | 10 |
|  | Sakardalla | 7 | 10 |
|  | Lokshura | 9 | 12.9 |
|  | Lmotit | 8 | 11.4 |
|  | Mpagas | 7 | 10 |
|  | Farakoren | 9 | 12.9 |
|  | Orotilkes | 7 | 10 |
|  | Korr | 8 | 11.4 |

Table S1: A table showing the details of all FGD participants
